# High PD-1 and CTLA-4 expression correlates with host immune suppression in patients and a mouse model infected with *Echinococcus multilocularis*

**DOI:** 10.1186/s13071-024-06511-2

**Published:** 2024-10-25

**Authors:** Ting Sun, Yi Yang, Yiwen Qiu, Tao Wang, Ming Yang, Shu Shen, Wentao Wang

**Affiliations:** Division of Liver Surgery, Department of General Surgery, West China Hospital, Sichuan University, Chengdu, People’s Republic of China

**Keywords:** Alveolar echinococcosis, *Echinococcus multilocularis*, Programmed cell death protein 1, Cytotoxic T lymphocyte-associated antigen 4, Immune suppression

## Abstract

**Background:**

Alveolar echinococcosis (AE), a fatal disease caused by *Echinococcus multilocularis*, often affects the liver, with tumor-like growth. However, the mechanism by which *E. multilocularis* evades host immune surveillance remains unclear.

**Methods:**

We collected liver specimens from hepatic alveolar echinococcosis (HAE) patients and established a mouse model of *E. multilocularis* infection. Immunofluorescence staining and flow cytometry were performed to analyze programmed cell death protein 1 (PD-1) and cytotoxic T lymphocyte associated antigen 4 (CTLA-4) expression in human samples, while flow cytometry and quantitative real-time polymerase chain reaction (PCR) were performed for similar analyses in mouse samples. Cell proliferation and protoscolex (PSC) killing assays were designed to explore how *E. multilocularis* induces host immunosuppression.

**Results:**

An inflammatory reaction band with high PD-1 and CTLA-4 expression was found in close liver tissue (CLT). The ratio of regulatory T cells (Tregs) was higher in CLT than in distant liver tissue (DLT), and Tregs in CLT tended to express higher levels of PD-1 and CTLA-4 than those in DLT from HAE patients. *Echinococcus multilocularis*-infected mice showed significantly elevated expression of PD-1 and CTLA-4 on splenocytes and peritoneal cells. PD-1/PD-L1 or CTLA-4 pathway blockade could relieve the immunosuppressive effects of Tregs from infected mice and enhance PSC killing by mouse splenocytes.

**Conclusions:**

*E. multilocularis* regulated the function of T cells via the PD-1/PD-L1- and CTLA-4-dependent pathways and subsequently evaded host immune attacks. These findings provide insights for investigating the pathogenic mechanism of AE.

**Graphical Abstract:**

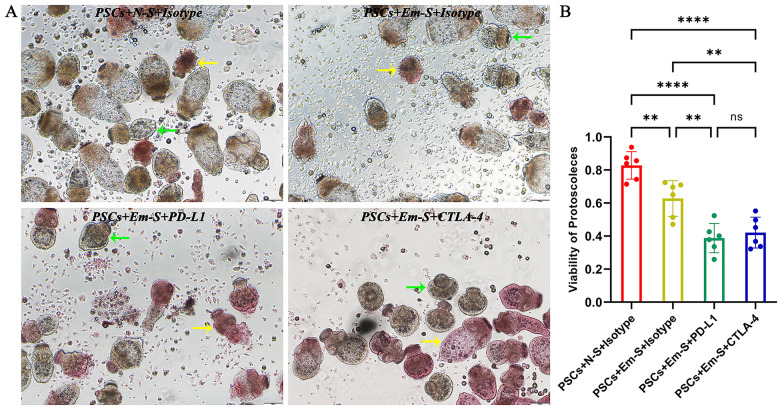

**Supplementary Information:**

The online version contains supplementary material available at 10.1186/s13071-024-06511-2.

## Background

Echinococcosis is a lethal zoonosis caused by *Echinococcus* and mainly includes two types: alveolar echinococcosis (AE) and cystic echinococcosis (CE). AE, which is caused by *Echinococcus multilocularis*, is less common but more severe and has a higher mortality rate [1]. Almost all primary AE lesions are in the liver and characterized by tumor-like infiltration, which frequently leads to invasion of vascular and biliary structures [2]. Previous studies showed that the mortality of untreated AE patients is > 90% within 10–15 years after diagnosis, and the average global burden of AE patients was estimated to be 666,434 disability-adjusted life years (DALYs) [1, 3].

Currently, the treatment of AE is mainly based on anti-infective drugs and surgery [4]. However, most patients are diagnosed and treated when AE progresses to the middle to late stages, which could impact treatment effectiveness [5]. Therefore, there is an urgent need to uncover the specific mechanism by which *E. multilocularis* invades the liver and identify potential targets for early intervention. On the one hand, available studies have demonstrated that the development and progression of many liver diseases are dependent on immune tolerance of the liver, which is mainly caused by exhaustion, anergy or apoptosis of antigen-specific T cells [6]. On the other hand, the specific immune response plays an important role in host defense against *E. multilocularis* infection, and host immune responses can inhibit the activity of AE lesions [7]. Therefore, programmed cell death protein 1 (PD-1)/programmed cell death ligand 1 (PD-L1), cytotoxic T lymphocyte-associated antigen 4 (CTLA-4) and other immune checkpoints that can mediate immune regulation and induce T-cell immunosuppression may play essential roles during *E. multilocularis* infection in humans.

PD-1, a member of the CD28/B7 family of immune checkpoints, is a type I transmembrane protein expressed on activated T cells, B cells, natural killer (NK) cells, etc. [8]. Some previous studies found that PD-1 expression was upregulated in *E. multilocularis*-infected hosts and might regulate immune cells against infection, which indicated that the PD-1 immune checkpoint may play a major role in facilitating immune escape during the parasite infection [9–12]. Furthermore, in a study on *Echinococcus granulosus* infection, investigators found that the levels of soluble PD-L1 and Th2 cytokines were much higher in CE patients than in healthy controls [13].

CTLA-4, which is also known as CD152, another member of the CD28/B7 family, is an inhibitory receptor mainly expressed on activated T cells and regulatory T cells (Tregs) [14]. A recent study has found that exhaustion-specific genes including CTLA-4 are upregulated in advanced-stage AE patients and may contribute to chronic parasite infection [12]. Furthermore, a study on malaria has demonstrated that Tregs impede protective immunity through CTLA-4 and that CTLA-4 blockade leads to significantly enhanced frequencies of Th1 and Th17 cells [15].

Although there have been some studies of PD-1 and CTLA-4 in parasitic infections, the roles played by PD-1 and CTLA-4 in the host immune response have not been fully elucidated in *E. multilocularis* infection. In this study, liver tissue specimens were collected from patients with hepatic alveolar echinococcosis (HAE), and a mouse model of *E. multilocularis* infection was established to observe the expression levels of PD-1 and CTLA-4. In vitro experiments were designed to further explore the mechanisms by which *E. multilocularis* induced host immunosuppression via PD-1 and CTLA-4 and achieved persistent infection.

## Methods

### Human subjects

All liver tissue specimens were collected from 10 HAE patients who underwent surgical treatment for the first time at West China Hospital of Sichuan University. Subjects with other hepatic diseases, immunological diseases, infectious diseases or pregnancy were excluded. Two experienced pathologists retrospectively analyzed surgical specimens, and the diagnosis of AE was confirmed by histological pathology. Liver tissues were collected and divided into two parts. One part was obtained close to the AE lesion (within 2 cm from the lesion) and was noted as “close liver tissue” (CLT), and the other part was obtained distant from the AE lesion (> 2 cm away from the lesion) and was noted as “distant liver tissue” (DLT) [16].

For multiplex immunofluorescence staining, the paraffinized liver sections were deparaffinized and rehydrated through a xylene/alcohol gradient and then subjected to antigen retrieval. The sections were incubated with CD4 antibody (Beyotime, AF1792) and PD-1 antibody (Proteintech, 66,220-1-Ig) or CD4 antibody (Proteintech, 67,786-1-Ig) and CTLA-4 antibody (Bioss, bs-1179R). The next day, the sections were incubated in corresponding fluorescence-labeled secondary antibodies and stained with DAPI (Beyotime, C1005). Thereafter, the sections were observed using a fluorescence microscope (Leica).

### Animal models of *Echinococcus multilocularis* infection

Specific pathogen-free (SPF) male Mongolian jirds (4 months old) and female BALB/c mice (6–8 weeks old) were purchased from Nanjing Qinglongshan Animal Breeding Center and Chengdu Dashuo Experimental Animal Co., Ltd., respectively. The animals were housed in an SPF animal room with a 12-h light/dark cycle and maintained on a standard rodent chow diet.

Protoscoleces (PSCs) were isolated from intraperitoneal lesions in infected Mongolian jirds as previously described [17]. In brief, the lesions were homogenized and filtered through nylon meshes with sterile phosphate-buffered saline (PBS, Gibco, 10,010,023). PSCs with > 95% viability confirmed by 0.1% eosin staining (Sigma-Aldrich, HT110116) were used for subsequent experiments. Mice were intraperitoneally inoculated with 1000 PSCs to establish the mouse model of *E. multilocularis* infection, while control group mice were intraperitoneally inoculated with the same volume of PBS.

### Isolation of human liver mononuclear cells and mouse primary cells

Human liver tissues were homogenized with RPMI 1640 (HyClone, SH30809.01) supplemented with neutral protease II (Invitrogen, 17,105,041), collagenase IV (Invitrogen, 17,104,019) and DNase (Invitrogen, 18,047,019). Mononuclear cells from liver tissues were isolated, hepatocytes were removed by centrifugation with 30% and 70% Percoll (Solarbio, P8370), and cells at the interface were collected [18].

Mouse primary peritoneal cells were isolated by lavage using prechilled PBS. The mouse spleen was removed aseptically and was homogenized. Furthermore, a CD4^+^ CD25^+^ Regulatory T-Cell Isolation Kit (Miltenyi Biotec, 130-091-041) was used to enrich Tregs from splenocytes according to the manufacturer’s instructions [19].

### Flow cytometry

Flow cytometry staining was performed using the antibodies listed in Table S1 according to the manufacturer’s protocol. For intracellular staining, a Fixation/Permeabilization Kit (BD Biosciences, 554,714) was used according to the manufacturer’s instructions. Fluorescence minus one (FMO) controls were used for gating wherever needed. Flow cytometry was performed on a BD FACSymphony A5 Cell Analyzer, and data were analyzed with FlowJo software (version 10, Tree Star, Inc).

### Quantitative real-time PCR

Total RNA was extracted and purified from peritoneal cells and splenocytes from mice. Reverse transcription reactions were carried out using the PrimeScript RT Reagent Kit with gDNA Eraser (TaKaRa, RR047A). Then, quantitative real-time PCR (polymerase chain reaction) was performed in a CFX Connect Real-Time PCR Detection System (Bio-Rad) with TB Green Premix Ex Taq II (TaKaRa, RR820A) according to the manufacturer’s instructions. The sequences of primers used in this study are listed in Table S2.

### Mouse T-cell proliferation assay

T-cell proliferation was measured using a carboxyfluorescein diacetate succinimidyl ester (CFSE) staining assay and evaluated by the expression of CD69. For the cell proliferation assay, splenocytes from uninfected control mice were first labeled with CFSE (5 μM, eBioscience, 65–0850-84). Labeled splenocytes were placed in sterile 24-well plates in RPMI 1640 medium containing 10% fetal bovine serum (FBS, Gibco, 10099141C) and 1% penicillin and streptomycin (Gibco, 15,140,122), with the following combinations: Tregs from uninfected control mice, Tregs from *E. multilocularis*-infected mice (90 days after inoculation, same below), Tregs from infected mice plus a PD-L1 antibody (1 μg/ml, BioXcell, BE0101) or Tregs from infected mice plus a CTLA-4 antibody (2 μg/ml, R&D Systems, MAB434). (Tregs were obtained by magnetic bead sorting using the CD4^+^ CD25^+^ Regulatory T-Cell Isolation Kit on splenocytes as previously described [19].) The ratio of splenocytes:Tregs was 16:1. Cells were stimulated with 2.5 μg/ml concanavalin A (ConA, Solarbio, C8110) for 4 days and then analyzed by flow cytometry as previously described.

### Killing of PSCs

PSCs were placed in sterile 48-well plates in RPMI 1640 medium containing 10% FBS and 1% penicillin and streptomycin with splenocytes from uninfected control mice or splenocytes from *E. multilocularis*-infected mice at a 1:2000 ratio. In addition, an anti-PD-L1 antibody (4 μg/ml, endotoxin < 0.001 EU/μg, BioXcell, CP168), anti-CTLA-4 antibody (4 μg/ml, endotoxin < 0.001 EU/μg, BioXcell, CP146) or isotype control antibody (4 μg/ml, endotoxin < 0.001 EU/μg, BioXcell, BP0083) was added to the co-culture system of PSCs and splenocytes from mice. PSCs and cells were co-cultured for 4 days. Ten visual fields for each well were randomly chosen, and mean values were calculated to evaluate PSC viability using 0.1% eosin staining under single-blinded conditions.

### Statistical analysis

The experimental results were expressed as the mean ± standard deviation (SD). Student’s t-test was used to compare two groups. One-way analysis of variance (ANOVA) plus Tukey’s multiple comparison test was used when there were more than two groups. Data with repeated measurements were analyzed using a mix-effects model with Sidak’s or Dunnett’s multiple comparison test. *P* < 0.05 was considered statistically significant in all experiments, and *p* values are expressed as follows: *p < 0.05; **p < 0.01; ***p < 0.001; ****p < 0.0001. Statistical analyses were performed using GraphPad Prism 9.0 (GraphPad Software, Inc.).

## Results

### PD-1/CTLA-4 expression was elevated in CLT from HAE patients

**Fig. 1:**
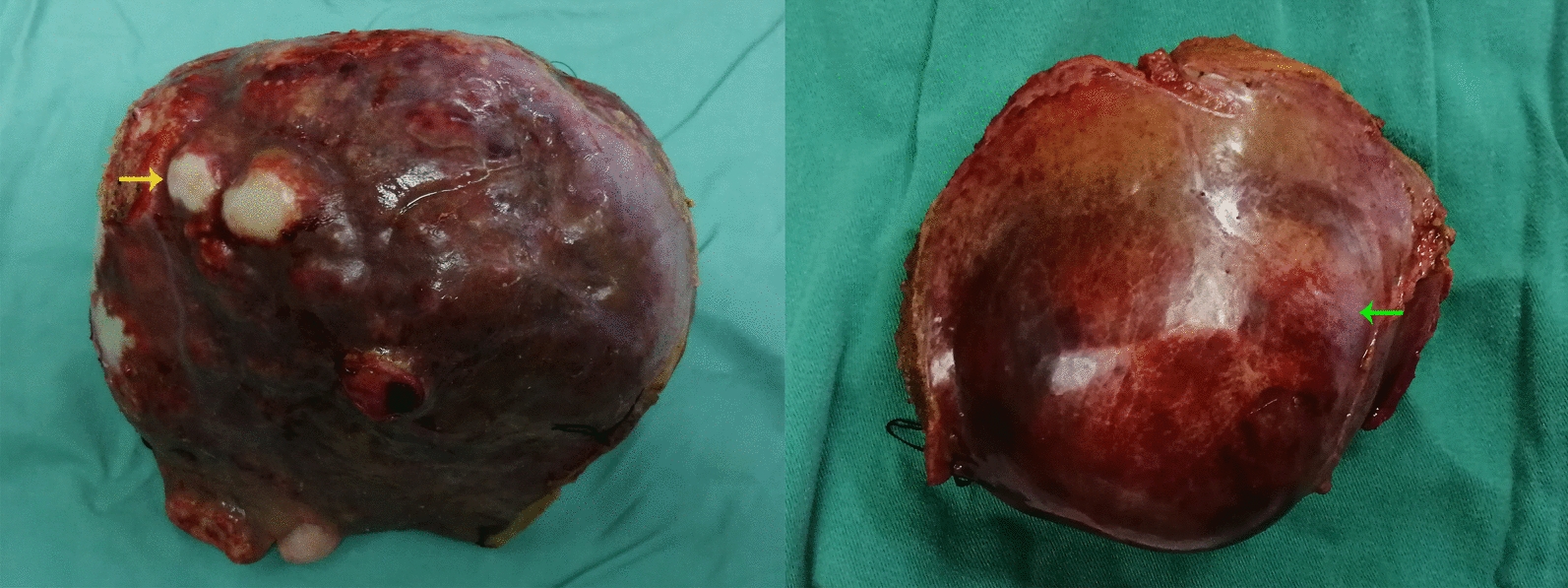
Gross appearance of liver specimens from hepatic alveolar echinococcosis patients. The yellow arrow indicates pale yellow hard nodules on the liver surface. The green arrow indicates the outline of the HAE lesion

**Fig. 2:**
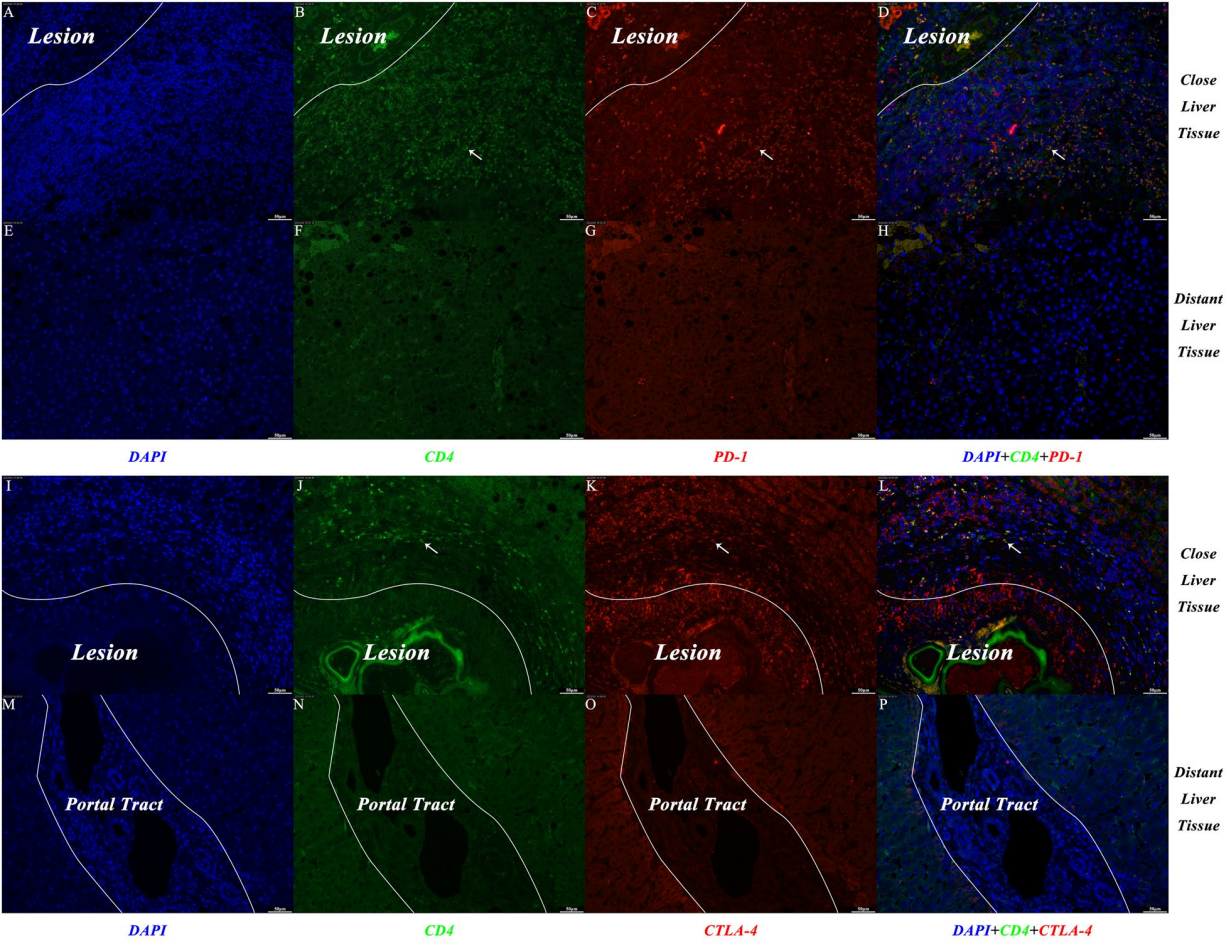
Representative images from immunofluorescence staining, with DAPI (blue), CD4 (green), PD-1/CTLA-4 (red) and merged images of close liver tissue and distant liver tissue from HAE patients. (**A**–**D** and **I**–**L**), close liver tissue; (**E**–**H** and **M**–**P**), distant liver tissue; (**A**, **E**,** I** and **M**), DAPI; (**B**, **F**, **J** and **N**), CD4; (**C** and **G**), PD-1; (**K** and **O**), CTLA-4; (**D**, **H**, **L** and **P**), merged. White arrows indicate CD4^+^ PD-1^+^ cells or CD4^+^ CTLA-4^+^ cells. White lines mark borders of parasitic lesions or portal tracts. Abbreviations: DAPI, 4’,6-diamidino-2-phenylindole; HAE, hepatic alveolar echinococcosis

We collected liver tissue samples from HAE patients, and representative gross specimens are shown in Fig. 1. To explore and compare the expression of PD-1 and CTLA-4 in liver tissue close to the HAE lesion and distant from the HAE lesion, multiplex immunofluorescence staining was performed on CLT and DLT, respectively. Figure 2A–H shows representative images displaying the expression of CD4 and PD-1 in the CLT and DLT. In contrast to DLTs, CD4^+^ immune cell aggregation and PD-1^+^ cell aggregation occurred in CLTs (Fig. 2B vs. F and Fig. 2C vs. G). CD4^+^ PD-1^+^ cell aggregation was also apparent in the CLT but absent in the DLT (Fig. 2D vs. H). Figure 2I–P shows the results of immunofluorescence staining displaying the expression of CD4 and CTLA-4. Similarly, CD4^+^ cell aggregation, CTLA-4^+^ cell aggregation and CD4^+^ CTLA-4^+^ cell aggregation were observed only in the CLT (Fig. 2J vs. N, Fig. 2K vs. O and Fig. 2L vs. P). These results implied that there is an evident inflammatory reaction band with high expression of PD-1 and CTLA-4 in CLT.

**Fig. 3:**
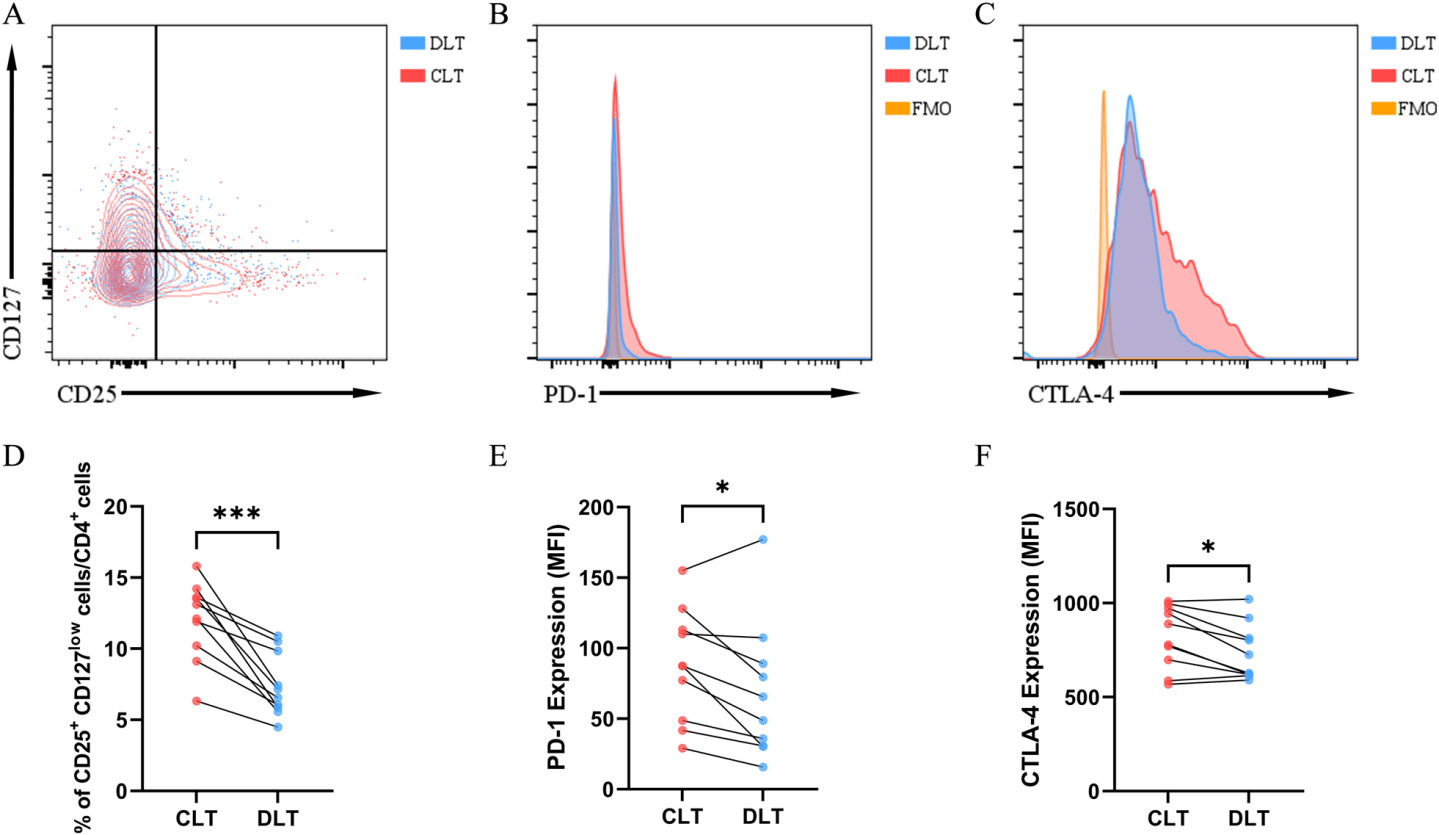
There was a larger proportion of CD4^+^ CD25^+^ CD127^low^ Tregs and higher expression of PD-1 and CTLA-4 on Tregs in CLT than in DLT from HAE patients. (**A**) Representative flow cytometry plots gated on CD3^+^ CD4^+^ CD8^−^ cells showing CD25 and CD127 expression in CLTs and DLTs. (**B**) Representative flow cytometry plots of Tregs showing PD-1 expression in the CLT and DLT. Yellow region represents background staining in FMO control. (**C**) Representative flow cytometry plots of Tregs showing CTLA-4 expression in the CLT and DLT. Yellow region represents background staining in FMO control. (**D**)Statistical comparison of the ratio of CD25^+^ CD127^low^ cells within CD4^+^ cells in the CLT and DLT from HAE patients (*n* = 10). (**E**) Statistical comparison of PD-1 MFI on Tregs in the CLT and DLT from HAE patients (*n* = 10). (**F**) Statistical comparison of CTLA-4 MFI on Tregs in CLT and DLT from HAE patients (*n* = 10). Data were analyzed using paired Student’s t-test. All data are presented as the mean ± standard deviation. *p < 0.05; ***p < 0.001. Abbreviations: Treg, regulatory T cell; CLT, close liver tissue; DLT, distant liver tissue; HAE, hepatic alveolar echinococcosis; FMO, fluorescence minus one; MFI, median fluorescence intensity

To further clarify which CD4^+^ T-cell type aggregated and highly expressed PD-1 and CTLA-4 in the CLT, flow cytometry was performed, and a representative gating strategy for CD4^+^ CD25^+^ CD127^low^ Tregs is shown in Figure S1. Figure S2 shows a larger proportion of CD4^+^ cells over all non-parenchymal cells in CLT than in DLT. Figure 3A shows CD25^+^ CD127^low^ Tregs gated on CD3^+^ CD4^+^ CD8^−^ cells, and Fig. 3D shows that the ratio of CD4^+^ CD25^+^ CD127^low^ Tregs/CD4^+^ T cells was significantly higher in the CLT than in the DLT. We also compared the expression of PD-1 and CTLA-4 on Tregs between CLTs and DLTs (Fig. 3B, C). Statistical results for median fluorescence intensity (MFI) indicated that Tregs in CLT expressed more PD-1 and CTLA-4 than those in DLT (Fig. 3E, F). Given that PD-1 and CTLA-4, as immune checkpoints, could negatively regulate immune responses and induce immune tolerance, it is highly likely that PD-1 and CTLA-4 on Tregs play vital roles in chronic infection with *E. multilocularis*.

### PD-1 and CTLA-4 were highly expressed on splenocytes and peritoneal cells in *E. multilocularis*-infected mice

**Fig. 4:**
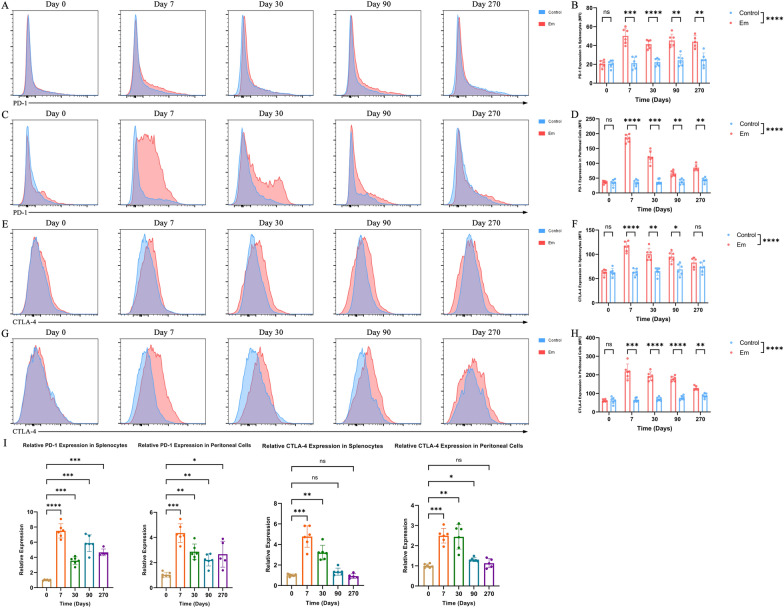
Higher expression of PD-1 and CTLA-4 on splenocytes and peritoneal cells in *Echinococcus multilocularis*-infected mice. (**A**) Representative flow cytometry plots of splenocytes showing PD-1 expression in infected and uninfected mice on days 0, 7, 30, 90 and 270 after inoculation. (**B**) Statistical comparisons of PD-1 MFI on splenocytes in infected and uninfected mice on days 0, 7, 30, 90 and 270 after inoculation (5—6 mice per group). (**C**) Representative flow cytometry plots of peritoneal cells showing PD-1 expression in infected and uninfected mice on days 0, 7, 30, 90 and 270 after inoculation. (**D**) Statistical comparisons of PD-1 MFI on peritoneal cells in infected and uninfected mice on days 0, 7, 30, 90 and 270 after inoculation (5—6 mice per group). (**E**) Representative flow cytometry plots of splenocytes showing CTLA-4 expression in infected and uninfected mice on days 0, 7, 30, 90 and 270 after inoculation. (**F**) Statistical comparisons of CTLA-4 MFI on splenocytes in infected and uninfected mice on days 0, 7, 30, 90 and 270 after inoculation (5—6 mice per group). (**G**) Representative flow cytometry plots of peritoneal cells showing CTLA-4 expression in infected and uninfected mice on days 0, 7, 30, 90 and 270 after inoculation. (**H**) Statistical comparisons of CTLA-4 MFI on peritoneal cells in infected and uninfected mice on days 0, 7, 30, 90 and 270 after inoculation (5—6 mice per group). (**I**) Quantitative real-time PCR detection of PD-1 and CTLA-4 expression on splenocytes and peritoneal cells on days 0, 7, 30, 90 and 270 after inoculation (5—6 mice per group). All splenocytes and peritoneal cells shown in plots (**A**–**H**) are gated on CD3^+^ CD4^+^ CD8^−^ cells. Data with repeated measurements were analyzed using a mix-effects model with Sidak’s or Dunnett’s multiple comparison test. All data are presented as the mean ± standard deviation and are representative of three independent experiments. *p < 0.05; **p < 0.01; ***p < 0.001; ****p < 0.0001; ns, *p* > 0.05. Abbreviations: *E. multilocularis/Em*, *Echinococcus multilocularis*; MFI, median fluorescence intensity; PCR, polymerase chain reaction; ns, not significant

A mouse model of *E. multilocularis* infection was successfully made as previously described [20]. Figure S3 shows representative appearances of intraperitoneal lesions from the mouse model and PSCs from intraperitoneal lesions under a light microscope. We isolated splenocytes and peritoneal cells from *E. multilocularis*-infected mice and uninfected control mice on days 0 (not yet infected) and 7, 30, 90 and 270 after inoculation. To assess changes in the expression of PD-1 and CTLA-4 caused by *E. multilocularis* infection, flow cytometry was performed, and a representative gating strategy for CD3^+^ CD4^+^ CD8^−^ T cells is shown in Figure S4. Figure 4A shows the expression of PD-1 on CD4^+^ T cells from splenocytes in *E. multilocularis*-infected mice and uninfected control mice on days 0, 7, 30, 90 and 270 after inoculation. We then compared the MFI of PD-1 on CD4^+^ T cells from splenocytes in infected and uninfected mice at various time points (Fig. 4B). Statistical analyses indicated that there were significant differences between the two groups, and CD4^+^ T cells from splenocytes in infected mice showed higher expression of PD-1 than those in uninfected mice on days 7, 30, 90 and 270 after inoculation. Figure 4C shows the expression of PD-1 on CD4^+^ T cells from peritoneal cells in infected and uninfected mice. Statistical analyses regarding the MFI of PD-1 indicated that CD4^+^ T cells from peritoneal cells in infected mice showed higher expression of PD-1 than those in uninfected mice on days 7, 30, 90 and 270 after inoculation (Fig. 4D). Similarly, Fig. 4E–H shows that the expression of CTLA-4 was higher in *E. multilocularis*-infected mice for both CD4^+^ T cells from splenocytes and peritoneal cells. Although the expression of PD-1 and CTLA-4 on CD4^+^ T cells from splenocytes and peritoneal cells was similarly variable, PD-1 and CTLA-4 expression showed significant differences between infected mice and uninfected mice and was significantly elevated at the vast majority of time points for infected mice.

To observe and validate PD-1 and CTLA-4 expression at the transcriptional level, quantitative real-time PCR was performed for splenocytes and peritoneal cells (Fig. 4I). Relative PD-1 expression was significantly elevated in splenocytes and peritoneal cells at all time points after inoculation. Similarly, relative CTLA-4 expression was significantly higher in splenocytes on days 7 and 30 after inoculation and was also higher in peritoneal cells on days 7, 30 and 90 after inoculation. The quantitative real-time PCR results showed the same trend as the above flow cytometry results. These findings indicated that *E. multilocularis* infection correlated with higher expression of PD-1 and CTLA-4 both systemically (on splenocytes) and locally (on peritoneal cells). *Echinococcus multilocularis* was highly likely to induce the expression of PD-1 and CTLA-4 on CD4^+^ T cells, which could promote host immune tolerance and favor *E. multilocularis* infection.

### Tregs from *E. multilocularis*-infected mice could significantly inhibit mouse T-cell activation and proliferation through the PD-1/PD-L1-dependent pathway and CTLA-4-dependent pathway

**Fig. 5:**
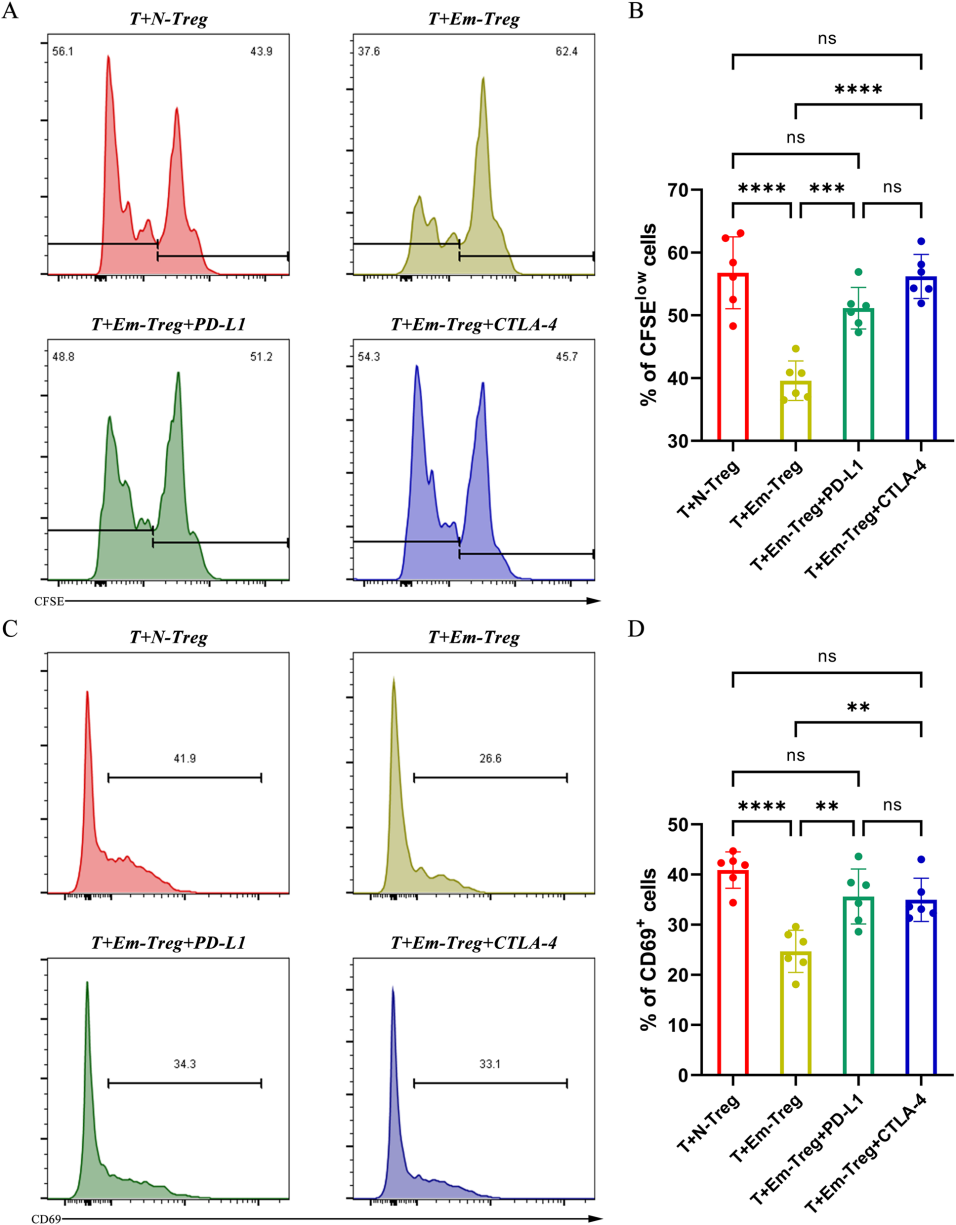
Inhibition of T-cell activation and proliferation by Tregs from *Echinococcus multilocularis*-infected mice through the PD-1/PD-L1- and CTLA-4-dependent pathways. (**A** and **B**) Representative flow cytometry plots showing the proliferation of CFSE-labeled T cells in four groups and statistical comparisons of the CFSE^low^ cell percentage among the groups. (**C** and **D**) Representative flow cytometry plots showing CD69 expression on T cells in four groups and statistical comparisons of the CD69^+^ cell percentage among the groups. All plots are gated on CD3^+^ CD25^−^ cells. “T” represents splenocytes from uninfected control mice (*n* = 6); “N-Treg” represents Tregs enriched from splenocytes from uninfected control mice (*n* = 6); “Em-Treg” represents Tregs enriched from splenocytes from *E. multilocularis*-infected mice (*n* = 6); “PD-L1” represents PD-L1 antibody; “CTLA-4” represents CTLA-4 antibody. Data were analyzed using one-way ANOVA plus Tukey’s multiple comparison test. All data are presented as the mean ± standard deviation and are representative of three independent experiments. **p < 0.01; ***p < 0.001; ****p < 0.0001; ns, *p* > 0.05. Abbreviations: Treg, regulatory T cell; CFSE, carboxyfluorescein diacetate, succinimidyl ester; *E. multilocularis*, *Echinococcus multilocularis*; ANOVA, analysis of variance

To clarify the roles of PD-1 and CTLA-4 during *E. multilocularis* infection, we designed a mouse T-cell proliferation assay in vitro. A representative gating strategy for CD3^+^ CD25^−^ T cells is shown in Figure S5. Figure 5A identifies proliferative T cells (CFSE low) and nonproliferative T cells (CFSE high) in each group, and Fig. 5B shows the statistical analysis of CFSE-low proliferating cells. We demonstrated that compared to Tregs from uninfected control mice, Tregs from *E. multilocularis*-infected mice could significantly inhibit mouse T-cell proliferation. When a PD-L1 antibody or CTLA-4 antibody was added, the percentage of CFSE-low proliferating cells increased, and T cells showed a restored ability to proliferate. Although the PD-L1 antibody or CTLA-4 antibody could cause corresponding immune checkpoint blockade, there were no significant differences in their abilities to restore T-cell proliferation.

In addition, we also observed the expression of the surface marker CD69 to assess the activation of T cells. Figure 5C shows CD69 expression on T cells in each group, and Fig. 5D shows a statistical comparison of the percentages of CD69^+^ T cells. Similar to CFSE-low T cells, the percentage of CD69^+^ T cells decreased in the presence of Tregs that were isolated from *E. multilocularis*-infected mice. When the PD-L1 antibody or CTLA-4 antibody was added, CD69 expression significantly increased, and the T cells showed a restored activation ability. Additionally, there were no significant differences in the abilities of the PD-L1 antibody and the CTLA-4 antibody to restore T-cell activation. Overall, compared to Tregs from uninfected control mice, Tregs induced by *E. multilocularis* infection could significantly inhibit mouse T-cell activation as well as proliferation in vitro, and immunosuppression was mediated through the PD-1/PD-L1-dependent pathway and CTLA-4-dependent pathway. When the concentration of the corresponding antibody was appropriate, blockade of PD-1/PD-L1 or CTLA-4 could effectively relieve immunosuppression and almost restore T-cell activation and proliferation abilities to the control levels.

### PD-1/PD-L1 or CTLA-4 blockade could significantly enhance the ability of *E. multilocularis*-infected mouse splenocytes to kill PSCs

**Fig. 6:**
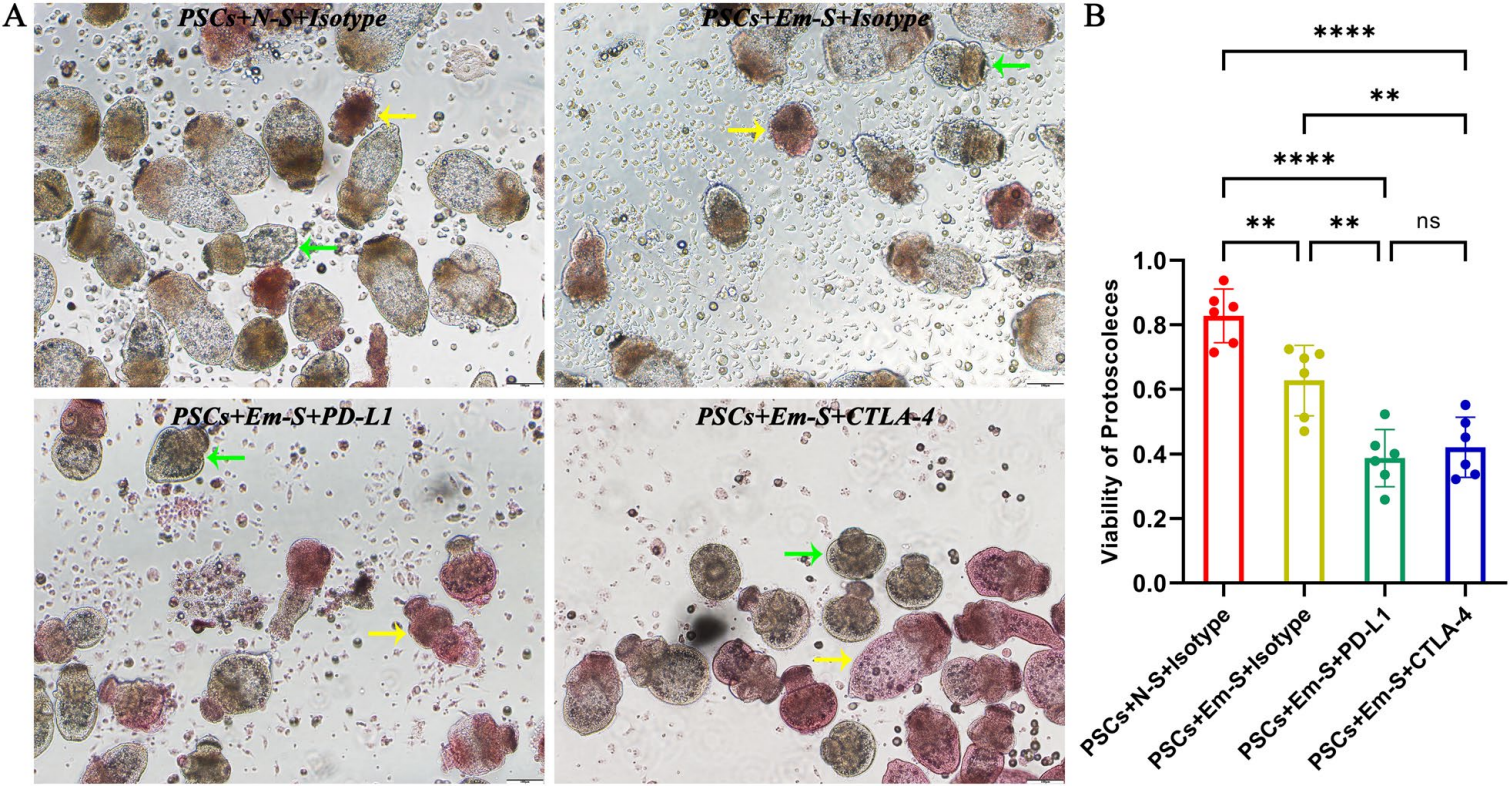
Enhanced ability of *Echinococcus multilocularis*-infected mouse splenocytes to kill PSCs caused by PD-1/PD-L1 or CTLA-4 blockade. (**A**) PSCs cultured in vitro under a light microscope in four groups (stained with 0.1% eosin, 100 × magnification). (**B**) Statistical comparisons of PSC viability among all groups. “PSCs” represents protoscoleces; “N-S” represents splenocytes from uninfected control mice (*n* = 6); “Em-S” represents splenocytes from *E. multilocularis*-infected mice (*n* = 6); “Isotype” represents isotype control antibody; “PD-L1” represents PD-L1 antibody; “CTLA-4” represents CTLA-4 antibody. The yellow arrow indicates dead PSCs completely stained red. The green arrow indicates unstained PSCs with high viability. Data were analyzed using one-way ANOVA plus Tukey’s multiple comparison test. All data are presented as the mean ± standard deviation and are representative of three independent experiments. **p < 0.01; ****p < 0.0001; ns, *p* > 0.05. Abbreviations: *E. multilocularis/Em*, *Echinococcus multilocularis*; PSCs, protoscoleces; ANOVA, analysis of variance

To further elucidate the effects of PD-1 and CTLA-4 on host immune responses against PSCs, we mimicked host immune cell-mediated killing of PSCs in vitro and compared the viability of PSCs under different conditions. Figure 6A shows the morphological appearances of PSCs after staining with eosin in each group, and Fig. 6B shows the statistical results for PSC viability. Compared to PSCs co-cultured with splenocytes from uninfected mice, PSCs co-cultured with splenocytes from infected mice showed lower viability, which was probably caused by immune memory. More importantly, splenocytes from infected mice showed a stronger ability to kill PSCs after addition of the PD-L1 antibody or CTLA-4 antibody. Given the results of a previous T-cell proliferation assay, we further demonstrated that PSCs could escape killing by host immune cells through a PD-1/PD-L1- and CTLA-4-dependent pathway. However, the enhanced killing ability caused by PD-1/PD-L1 blockade, and that caused by CTLA-4 blockade, showed no significant differences.

## Discussion

AE lesions often present with tumor-like growth in the human liver, invade the intrahepatic vasculature and tend to develop extrahepatic metastases, which contributes to the difficulty of treatment [21]. Although radical resection surgery combined with albendazole has long been the most commonly used therapeutic strategy [4], approximately a quarter of HAE patients have not been recommended to receive surgery at the time of diagnosis [22]. Therefore, a thorough understanding of the pathogenic mechanisms of HAE and further exploration of novel therapeutic targets are important.

Recently, several studies have reported that immune checkpoints, including PD-1, CTLA-4, LAG-3, TIM-3, TIGIT, etc., were associated with immune tolerance and immune escape in parasitic infections [15, 23–25]. For *E. multilocularis* infection, some basic studies have shown that changes in the host’s immune status are important factors affecting the progression and outcome of the disease [17, 26–29]. *Echinococcus multilocularis* can induce NK cell exhaustion [28] and M2 macrophage polarization [30] to achieve immune escape. Investigators also found that some excretory proteins of *E. multilocularis* could regulate the host immune system and induce host immune tolerance [29]. However, the mechanisms by which *E. multilocularis* induces immunosuppression and evades immune surveillance are incompletely understood in AE. In the present study, we focused on the roles of PD-1 and CTLA-4 during *E. multilocularis* infection and demonstrated that the proliferation and killing capacity of host immune cells might be negatively regulated by these two checkpoints.

The inflammatory reaction band appearing around the lesion was the major histopathological signature of AE, while the center of the lesion often developed caseous necrosis due to ischemia. The inflammatory band where CD4^+^ immune cell aggregation occurred could be observed in the CLT. On this basis, we localized PD-1 and CTLA-4 to aggregated CD4^+^ T cells in the CLT by immunofluorescence studies. Furthermore, we used flow cytometry to confirm that there were more CD4^+^ CD25^+^ CD127^low^ Tregs in the CLT than in the DLT and that the Tregs in the CLT expressed more PD-1 and CTLA-4. Because host-*E. multilocularis* immune response was mainly observed in the inflammatory reaction band of the CLT, it was reasonable to speculate that PD-1 and CTLA-4 were involved in host immune regulation during chronic *E. multilocularis* infection.

It was difficult to compare the liver tissue of HAE patients with the liver tissue of cured patients because most HAE patients were diagnosed at a late stage. Taking ethical considerations into account, we decided to employ a mouse model of *E. multilocularis* for further investigation. We compared the immune status and immune checkpoint expression of infected mice with those of uninfected mice at five different time points. We found elevated perilesional expression of PD-1 in peritoneal cells from infected mice, in agreement with previous studies [9, 12]. Furthermore, we also found elevated expression of PD-1 associated with systemic regulation of immunity by splenocytes from infected mice. For CTLA-4, we obtained results similar to those obtained for PD-1, and no relevant studies have focused on the role of CTLA-4 in a mouse model of *E. multilocularis* infection. Notably, the increase in PD-1 and CTLA-4 expression on splenocytes and peritoneal cells from infected mice was even more pronounced in the early stage of infection (7 days and 30 days after inoculation) based on the results of flow cytometry and PCR. This suggested that to establish chronic infection, *E. multilocularis* could induce upregulation of PD-1 and CTLA-4 and lead to an immunosuppressive state in the host in the early stage of infection both systemically and locally, which might partly explain the mechanisms of early immunosuppression in *E. multilocularis* infection [31]. Furthermore, the remarkably increased expression of PD-1 and CTLA-4 may be related to activated antigen-specific T cells and involved in protecting against severe immunopathology during the acute phase of infection [32].

As previous portions of this study had revealed that PD-1 and CTLA-4 on Tregs are highly likely to play key roles during *E. multilocularis* infection, we designed a T-cell proliferation assay and explored the regulation of host T cells by Tregs. We found that PD-1/PD-L1 blockade almost reversed the impaired proliferation of T cells caused by Tregs from infected mice to the control level in vitro. Notably, this study provides the first report that CTLA-4 blockade has a similar function as PD-1/PD-L1 blockade and can also significantly relieve the inhibition of T-cell proliferation caused by Tregs from *E. multilocularis*-infected mice. Previously, researchers determined the contribution of CTLA-4 to the regulation of immune cell responses and mechanisms of immunosuppression during other parasitic infections, such as *Plasmodium* [15]. Considering the role played by CTLA-4 in *E. multilocularis* infection, we propose that there is much need for further research on AE diagnosis and treatment.

To intuitively assess the effects of PD-1/PD-L1 and CTLA-4 blockade on host immune responses against PSCs, we co-cultured PSCs with splenocytes and designed a PSC killing experiment. Together with the above cell proliferation assay, our results revealed that host immune tolerance caused by *E. multilocularis* infection was PD-1/PD-L1 and CTLA-4 dependent. The co-cultivation of *E. multilocularis* and host cells was first described by Kanazawa et al. in 1993 [33]. During the past 30 years, only a few studies have reported the hepatocyte-killing effect of *E. multilocularis* in vitro because of the harsh in vitro culture conditions of *E. multilocularis* [34–36]. Liu et al. demonstrated that crocin could result in structural damage to *E. multilocularis* in vitro and might be developed as a new drug against AE [35]. Schubert et al. found that BI 2536, a Plk1 inhibitor, could induce germinative cell killing and suggested that it was a promising compound for AE chemotherapy [36]. Immune checkpoint-targeted drugs need to be further studied and might be applied to treat AE or other parasitic diseases in the future.

It is currently widely believed that T cell-mediated immune regulation, especially the host immunosuppression mediated by CD4 + T cells, is closely related to chronic *E. multilocularis* infection [37]. In the early stages of *E. multilocularis* infection, the host's immune response may be characterized by a mixed Th1/Th2 profile, whereas in the middle to late stages of infection, the host's immune response is predominantly characterized by a Th2/Treg profile [37, 38]. Th2 cells and a range of Tregs can establish a microenvironment that possesses an immunosuppressive effect by releasing a variety of cytokines, including interleukin (IL)-4, IL-5, IL-10, IL-13 and transforming growth factor β1 (TGF-β1). They can impede the development of antigen-presenting cells or induce T cells to adopt an immunoregulatory profile, which is commonly referred to as “infectious tolerance” [39]. The immunological tolerance phenomena involving the aforementioned components have been demonstrated in AE patients and animal models of *E. multilocularis* infection [40], and the findings are also supported by the data from this study. A clinical study also found that the peripheral blood of AE patients is predominantly characterized by a mixed cytokine profile of Th2/Th17/Treg [41]. The levels of Th2-associated cytokines in the serum, such as IL-23 and IL-5, can to some extent be utilized to assess the disease progression of AE [41]. The relationship between *E. multilocularis* infection and the host's immune response is highly complex, necessitating more in-depth research to further elucidate it.

## Conclusions

Our study found that PD-1 and CTLA-4 might be essential immune checkpoints correlating with host immune suppression in *E. multilocularis* infection. We speculated that *E. multilocularis* might be capable of evading the host immune response via the PD-1/PD-L1-dependent pathway and CTLA-4-dependent pathway. This process was probably associated with the expansion of host Tregs. These findings could potentially highlight new prospects for the diagnosis and immunotherapy of parasitic diseases.

## Supplementary Information

Supplementary file 1: Figure S1. Representative gating strategy for CD3^+^ CD4^+^ CD8^-^ CD25^+^ CD127^low^ T cells from human liver tissues. (A)–(F) Representative gating strategies for CLT. (G)–(L) Representative gating strategies for DLT. Abbreviations: CLT, close liver tissue; DLT, distant liver tissue; FVS700, fixable viability stain 700 (BD Horizon).

Supplementary file 2: Figure S2. Statistical comparison of the percentage of CD3^+^ CD4^+^ CD8^-^ cells over all non-parenchymal cells in the CLT and DLT from HAE patients (*n* = 10). Data were analyzed using paired Student’s t-test. **p < 0.01. Abbreviations: CLT, close liver tissue; DLT, distant liver tissue; HAE, hepatic alveolar echinococcosis.

Supplementary file 3: Figure S3. Representative macroscopic appearances of the mouse model with *Echinococcus multilocularis* infection and PSCs isolated from the model under a light microscope. (A) Macroscopic appearance of an *E. multilocularis*-infected mouse on day 90 after inoculation and its intraperitoneal lesions. The yellow arrow indicates AE lesions on the liver surface. The green arrow indicates AE lesions in the abdominal cavity. (B) Macroscopic appearance of an *E. multilocularis*-infected mouse on day 270 after inoculation and its intraperitoneal lesions. The yellow arrow indicates AE lesions on the liver surface. The green arrow indicates AE lesions in the abdominal cavity. (C) PSCs isolated from the AE lesions of the mouse model under a light microscope (unstained, 100 × magnification). (D) PSCs isolated from the AE lesions of the mouse model under a light microscope (stained with 0.1% eosin, 100× magnification). The yellow arrow indicates dead PSCs completely stained red. The green arrow indicates unstained PSCs with high viability. Abbreviations: PSCs, protoscoleces; AE, alveolar echinococcosis.

Supplementary file 4: Figure S4. Representative gating strategy for CD3^+^ CD4^+^ CD8^-^ T cells from mice. Abbreviations: FVS700, fixable viability stain 700 (BD Horizon).

Supplementary file 5: Figure S5. Representative gating strategy for CD3^+^ CD25^-^ T cells from mice. Abbreviations: FVS700, fixable viability stain 700 (BD Horizon).

Supplementary file 6.

Supplementary file 7.

## Data Availability

The data analyzed during the current study are available from the corresponding author upon reasonable request.
