# Supplementary figures and images for: High PD-1 and CTLA-4 expression correlates with host immune suppression in patients and a mouse model infected with Echinococcus multilocularis
Source: Parasit Vectors. 2024 Oct 25;17:437. doi: 10.1186/s13071-024-06511-2 (PMC11515268; doi:10.1186/s13071-024-06511-2)

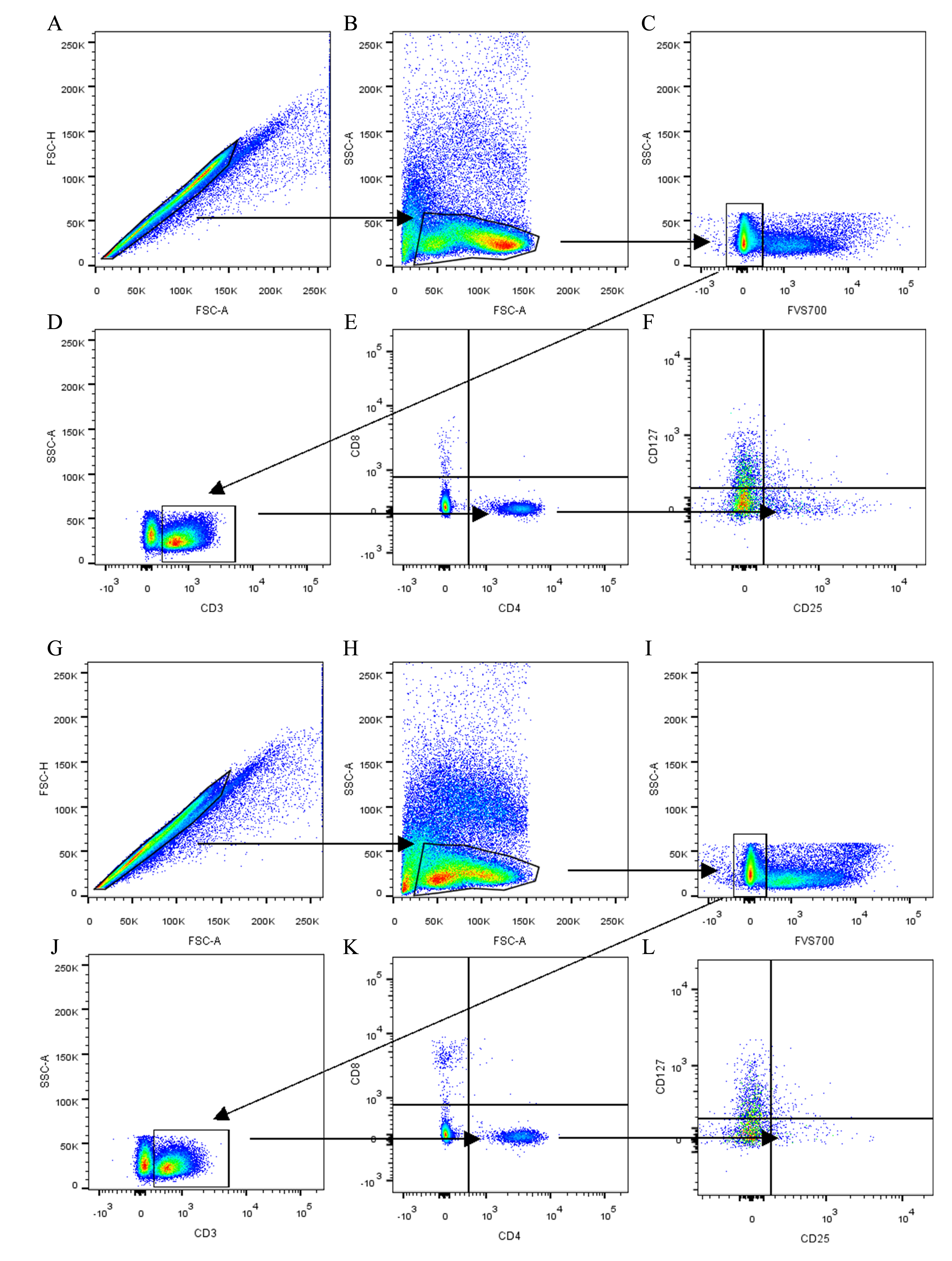

Supplement: Supplementary file 1 — Figure S1. Representative gating strategy for CD3+ CD4+ CD8- CD25+ CD127low T cells from human liver tissues. (A)–(F) Representative gating strategies for CLT. (G)–(L) Representative gating strategies for DLT. Abbreviations: CLT, close liver tissue; DLT, distant liver tissue; FVS700, fixable viability stain 700 (BD Horizon). [file 13071_2024_6511_MOESM1_ESM.tif]

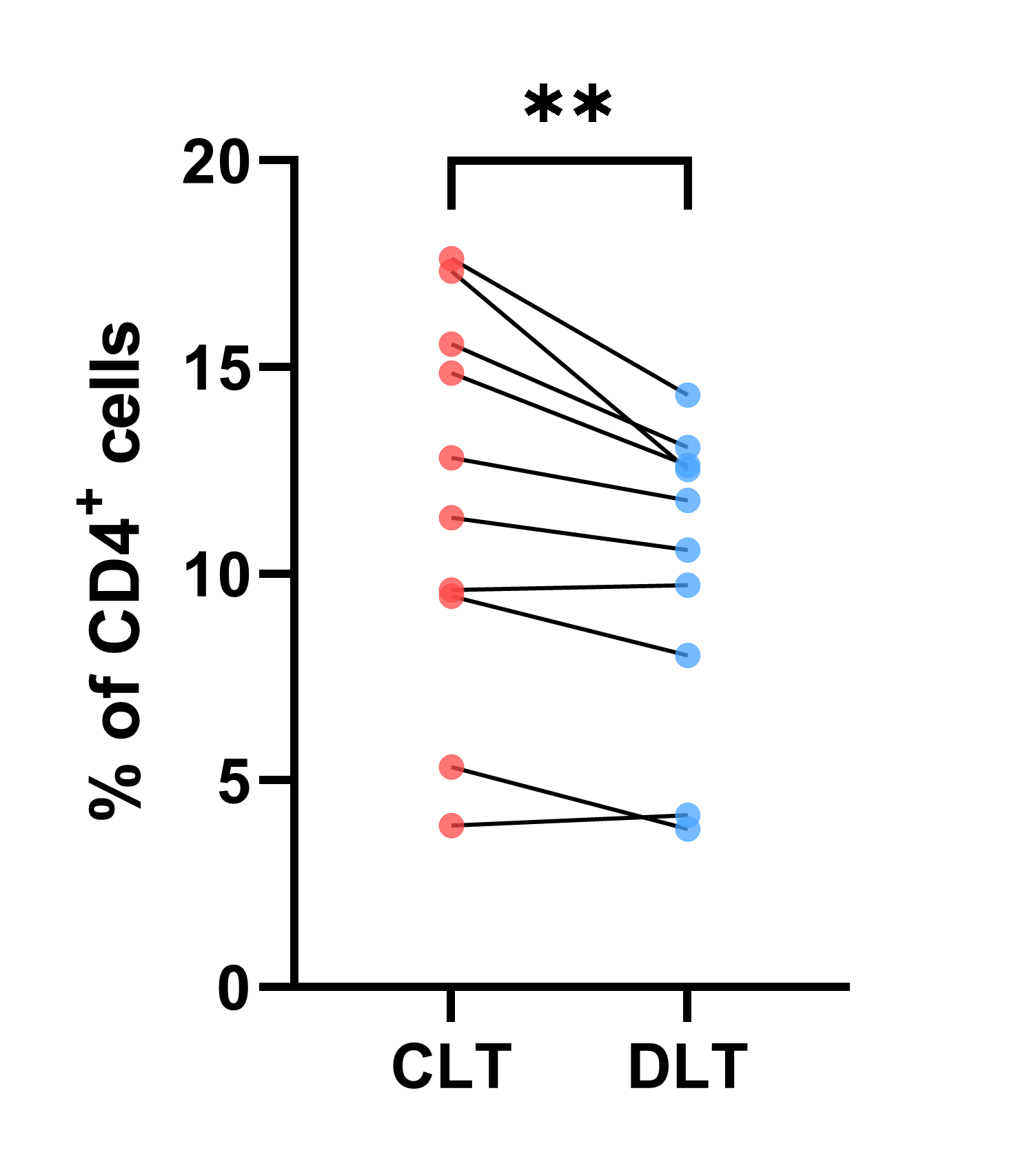

Supplement: Supplementary file 2 — Figure S2. Statistical comparison of the percentage of CD3+ CD4+ CD8- cells over all non-parenchymal cells in the CLT and DLT from HAE patients (n = 10). Data were analyzed using paired Student’s t-test. **p < 0.01. Abbreviations: CLT, close liver tissue; DLT, distant liver tissue; HAE, hepatic alveolar echinococcosis. [file 13071_2024_6511_MOESM2_ESM.tif]

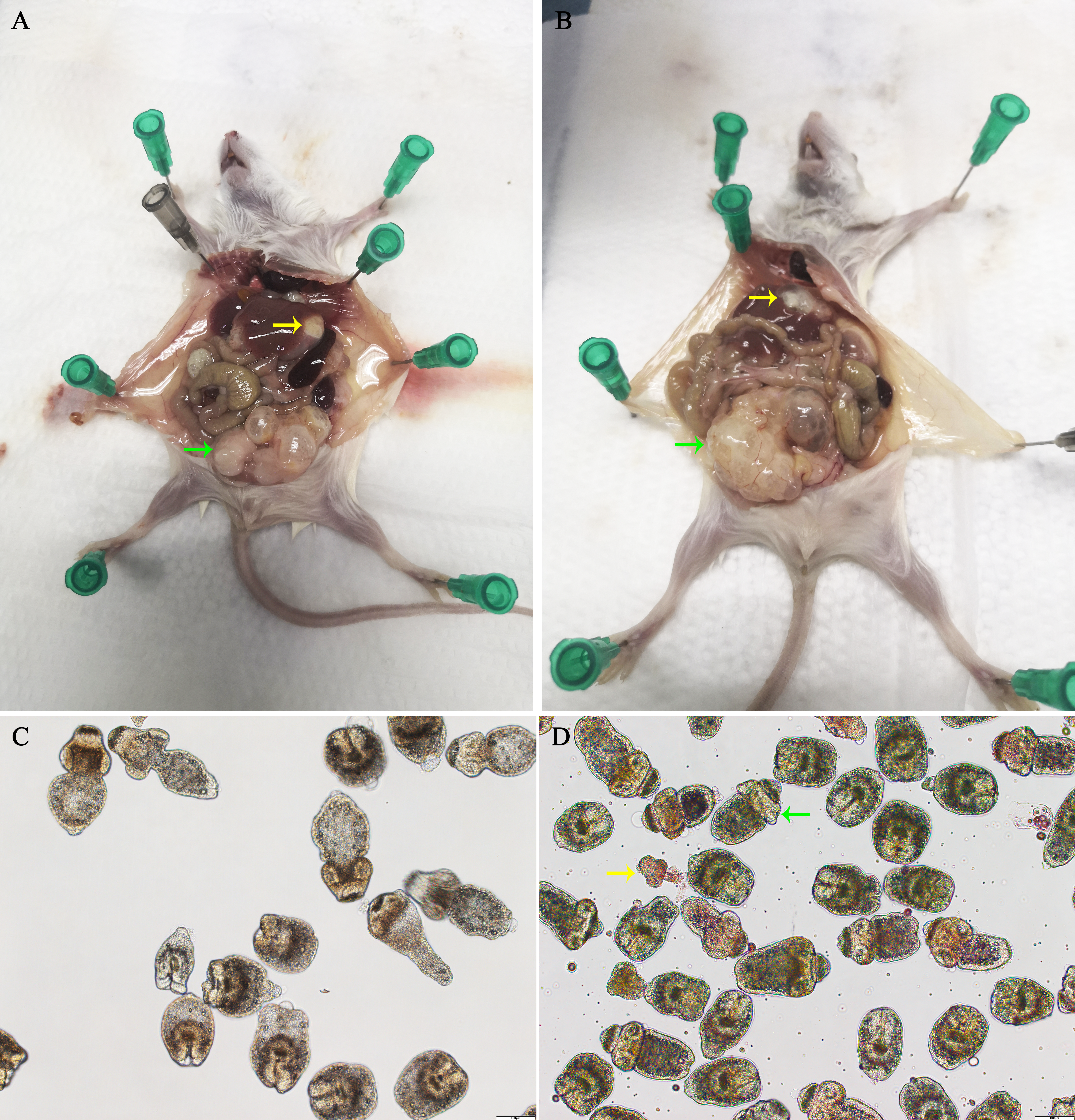

Supplement: Supplementary file 3 — Figure S3. Representative macroscopic appearances of the mouse model with Echinococcus multilocularis infection and PSCs isolated from the model under a light microscope. (A) Macroscopic appearance of an E. multilocularis-infected mouse on day 90 after inoculation and its intraperitoneal lesions. The yellow arrow indicates AE lesions on the liver surface. The green arrow indicates AE lesions in the abdominal cavity. (B) Macroscopic appearance of an E. multilocularis-infected mouse on day 270 after inoculation and its intraperitoneal lesions. The yellow arrow indicates AE lesions on the liver surface. The green arrow indicates AE lesions in the abdominal cavity. (C) PSCs isolated from the AE lesions of the mouse model under a light microscope (unstained, 100 × magnification). (D) PSCs isolated from the AE lesions of the mouse model under a light microscope (stained with 0.1% eosin, 100× magnification). The yellow arrow indicates dead PSCs completely stained red. The green arrow indicates unstained PSCs with high viability. Abbreviations: PSCs, protoscoleces; AE, alveolar echinococcosis. [file 13071_2024_6511_MOESM3_ESM.tif]

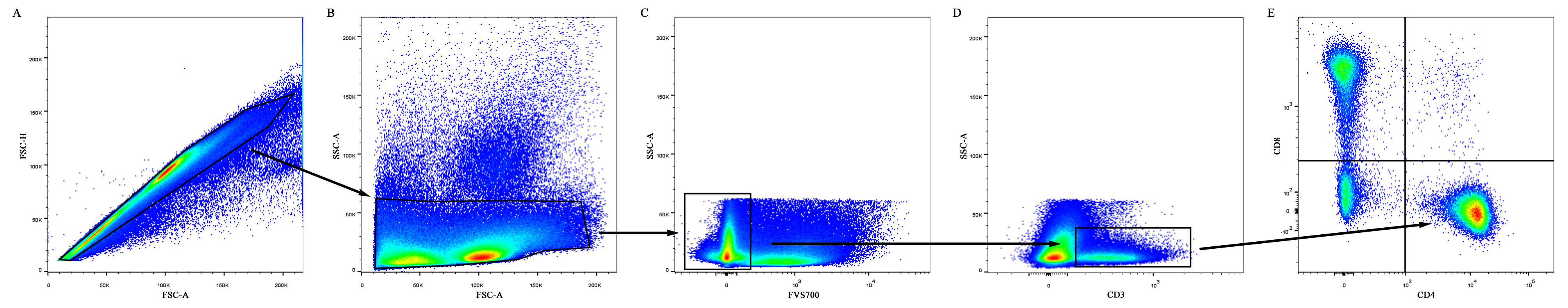

Supplement: Supplementary file 4 — Figure S4. Representative gating strategy for CD3+ CD4+ CD8- T cells from mice. Abbreviations: FVS700, fixable viability stain 700 (BD Horizon). [file 13071_2024_6511_MOESM4_ESM.tif]

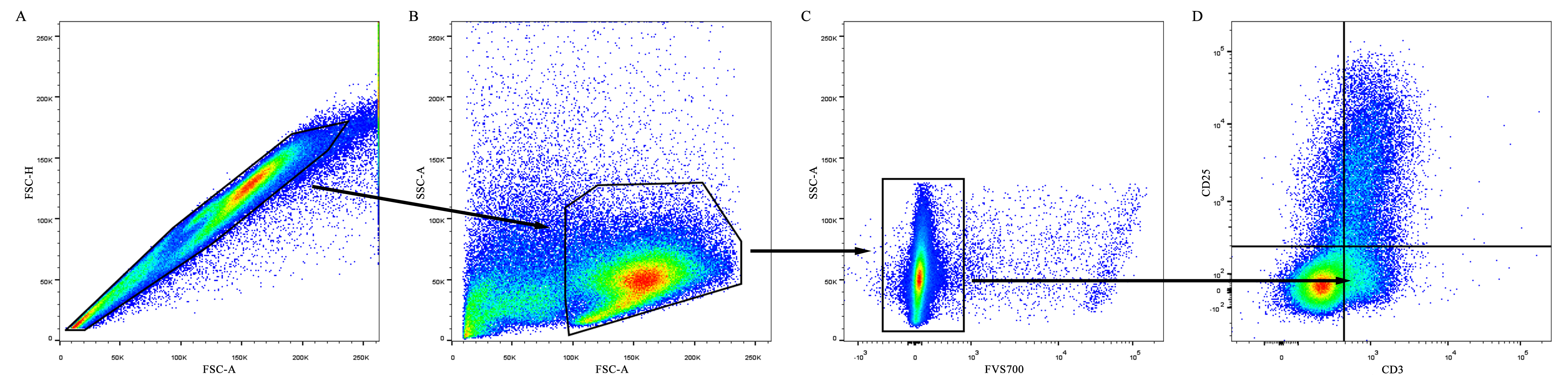

Supplement: Supplementary file 5 — Figure S5. Representative gating strategy for CD3+ CD25- T cells from mice. Abbreviations: FVS700, fixable viability stain 700 (BD Horizon). [file 13071_2024_6511_MOESM5_ESM.tif]
