# Supplementary material for: High PD-1 and CTLA-4 expression correlates with host immune suppression in patients and a mouse model infected with Echinococcus multilocularis
Source: Parasit Vectors. 2024 Oct 25;17:437. doi: 10.1186/s13071-024-06511-2 (PMC11515268; doi:10.1186/s13071-024-06511-2)
Supplement: Supplementary file 6 [file 13071_2024_6511_MOESM6_ESM.docx]

**Table S1. Antibodies for flow cytometry**

| **Reagent** | **Clone** | **Source** | **Catalog Number** |
| --- | --- | --- | --- |
| Human Fc Block | Fc1 | BD Biosciences | 564219 |
| FITC anti-human CD3 | OKT3 | BioLegend | 317306 |
| APC anti-human CD4 | RPA-T4 | BioLegend | 300514 |
| PerCP-Cy5.5 anti-human CD8 | SK1 | BioLegend | 344709 |
| PE-Cy7 anti-human CD25 | M-A251 | BD Biosciences | 557741 |
| BUV737 anti-human CD127 | HIL-7R-M21 | BD Biosciences | 564300 |
| BV421 Anti-Human CD279 (PD-1) | MIH4 | BD Biosciences | 564323 |
| PE anti-human CD152 (CTLA-4) | L3D10 | BioLegend | 349906 |
| Anti-mouse CD16/CD32 | 2.4G2 | BD Biosciences | 553141 |
| FITC anti-mouse CD3 | 17A2 | BioLegend | 100203 |
| APC anti-mouse CD4 | GK1.5 | BioLegend | 100412 |
| PerCP-Cy5.5 anti-mouse CD8a | 53-6.7 | BD Biosciences | 551162 |
| APC anti-mouse CD25 | PC61 | BD Biosciences | 557192 |
| BV421 anti-mouse CD279 (PD-1) | 29F.1A12 | BioLegend | 135221 |
| PE anti-mouse CD152 (CTLA-4) | UC10-4B9 | BioLegend | 106305 |
| BV786 anti-Mouse CD69 | H1.2F3 | BD Biosciences | 564683 |
| Fixable Viability Stain 700 | - | BD Biosciences | 564997 |
