# Supplementary material for: High PD-1 and CTLA-4 expression correlates with host immune suppression in patients and a mouse model infected with Echinococcus multilocularis
Source: Parasit Vectors. 2024 Oct 25;17:437. doi: 10.1186/s13071-024-06511-2 (PMC11515268; doi:10.1186/s13071-024-06511-2)
Supplement: Supplementary file 7 [file 13071_2024_6511_MOESM7_ESM.docx]

**TableS2. Primer sequences for quantitative real-time PCR**

| **Gene** | **GenBank Accession** | **Forward Primer** | **Reverse Primer** |
| --- | --- | --- | --- |
| β-actin | NM_007393 | GGCTGTATTCCCCTCCATCG | CCAGTTGGTAACAATGCCATGT |
| PD-1 | NM_008798 | ACCCTGGTCATTCACTTGGG | CATTTGCTCCCTCTGACACTG |
| CTLA-4 | NM_009843 | GCTTCCTAGATTACCCCTTCTGC | CGGGCATGGTTCTGGATCA |
